# Overcoming the Pitfalls of Computing Reaction Selectivity from Ensembles of Transition States

**DOI:** 10.1021/acs.jpclett.4c01657

**Published:** 2024-07-11

**Authors:** Ruben Laplaza, Matthew D. Wodrich, Clemence Corminboeuf

**Affiliations:** † Laboratory for Computational Molecular Design, Institute of Chemical Sciences and Engineering, École Polytechnique Fédérale de Lausanne (EPFL), 1015 Lausanne, Switzerland; ‡ National Center for Competence in Research-Catalysis (NCCR-Catalysis), École Polytechnique Fédérale de Lausanne, 1015 Lausanne, Switzerland

## Abstract

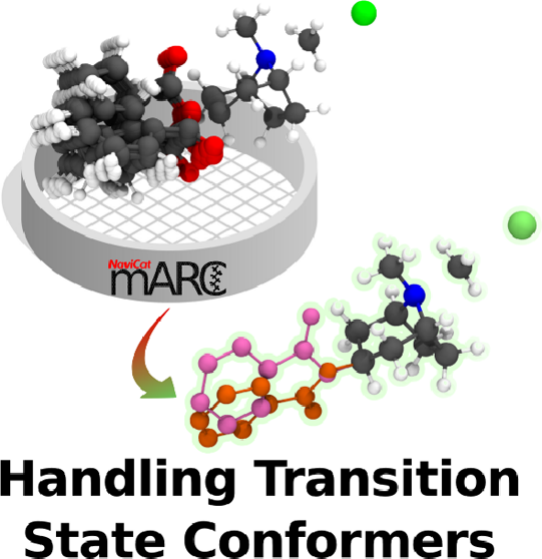

The prediction of reaction selectivity is a challenging task for computational chemistry, not only because many molecules adopt multiple conformations but also due to the exponential relationship between effective activation energies and rate constants. To account for molecular flexibility, an increasing number of methods exist that generate conformational ensembles of transition state (TS) structures. Typically, these TS ensembles are Boltzmann weighted and used to compute selectivity assuming Curtin-Hammett conditions. This strategy, however, can lead to erroneous predictions if the appropriate filtering of the conformer ensembles is not conducted. Here, we demonstrate how any possible selectivity can be obtained by processing the same sets of TS ensembles for a model reaction. To address the burdensome filtering task in a consistent and automated way, we introduce *marc*, a tool for the modular analysis of representative conformers that aids in avoiding human errors while minimizing the number of reoptimization computations needed to obtain correct reaction selectivity.

Relying on computational methods, such as density functional theory (DFT), to accurately predict reaction selectivity remains a key challenge for *in silico* catalyst design.^1−5^ Small errors in computed transition state (TS) energies, even those below chemical accuracy (1 kcal/mol), can result in a reversal of predicted selectivity^6^ due to the exponential relationship between effective activation energies and rate constants.^7−12^ Dealing with these accuracy issues can further be complicated when large and flexible functional groups used to impart asymmetry through noncovalent interactions are present,^13−15^ as these larger systems are likely to adopt multiple TS conformations.

Computational approaches for estimating selectivity often resort to choosing one (or a handful) of relevant conformations derived from either “chemical intuition” or experimental evidence. The relative free energies of the presumed reaction pathways are then computed and the resulting selectivity estimated.^16−18^ While this computationally inexpensive approach may work for simple systems, it becomes increasingly tricky for larger species and cannot be generalized to large pools of catalysts.^19−23^ On the other extreme, (*ab initio*) molecular dynamics combined with enhanced sampling techniques such as metadynamics^24^ or replica exchange^25−28^ (which may be powered by machine learning potentials^28−32^) can provide full conformational landscapes that would yield accurate selectivity predictions. In practice, however, such approaches are generally too computationally demanding, either in terms of directly modeling the system over long time frames, or in generating the amount of training data needed to create ML models, and are thus limited to smaller systems.^30,32−34^

One pragmatic approach for determining selectivity from DFT data is to assume a system operates under Curtin-Hammett conditions.^35^ In such cases, full conformational sampling of TS structures can be undertaken, and the resulting energies weighted to obtain final product ratios.^17,18,36−41^ The popularity of this “Curtin-Hammett Conformational Sampling” (CHCS) method has fostered an increasing number of tools that rely on rotamer libraries,^42−45^ inexpensive potentials combined with enhanced sampling techniques^46−48^ as popularized by the CREST program^49−53^ or distance geometry methods^54−59^ to generate conformational ensembles of a molecule. These approaches provide more complete pictures of selectivity but also require additional computations. As an example of the importance of conformational degrees of freedom, we recently demonstrated how on-the-fly conformational sampling can be used to accurately model enantioselectivity for a diverse set of catalysts with reduced human intervention.^59−61^

When using any of the aforementioned approaches for conformer sampling, particularly the automated variants, inadequate handling of the TS ensembles can lead to significant errors in selectivity estimations. This arises primarily due to two situations: (1) the counting of multiple equivalent transition states (Repeated conformers, Figure 1) and (2) not distinguishing interconvertible and noninterconvertible pathways (Non-interconvertible conformers, Figure 1). Here, we highlight potential pitfalls of using the CHCS strategy by demonstrating how processing the same ensemble of computed TS conformers in various ways leads to virtually any selectivity prediction, even for a simple organic reaction. We then introduce *marc*, a tool for the ***m***odular ***a***nalysis of ***r***epresentative ***c***onformers that improves selectivity predictions by untangling conformational ensembles through automated conformer classification and filtering.

**Figure 1:**
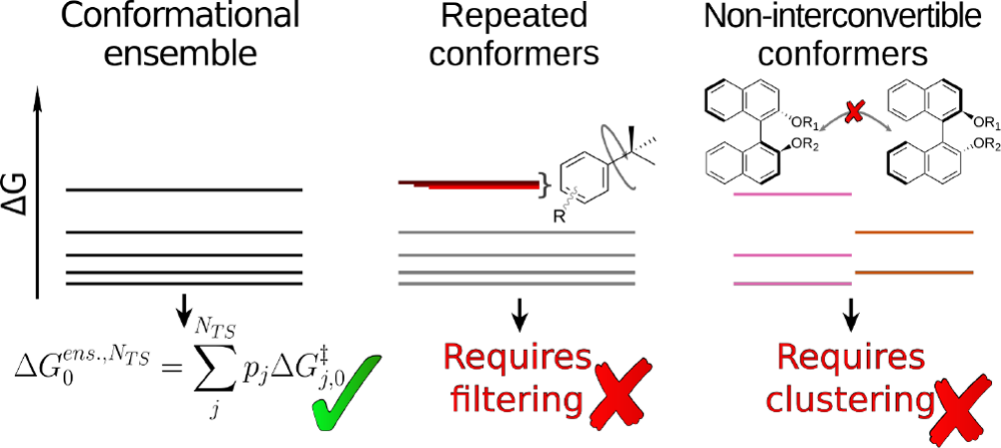
Schematic representation of the two concealed error sources in transition state conformer weighting.

*Concealed Error Sources in Transition State Conformer Weighting.* “Repeated conformer” errors arise when the same (or fundamentally identical) TSs present within an ensemble are counted multiple times. Such errors have different effects depending on how the selectivity is determined. In Boltzmann weighting, for instance, a repeated high energy TS can artificially raise the TS barrier height toward that product. On the other hand, if selectivity is assessed directly from rate constants, then a repeated low energy TS can artificially lower the barrier toward that product. In theory, such errors are easily avoided by manually filtering redundant species. Automation, however, introduces its own set of problems as small numerical discrepancies in bond lengths/angles and/or energies cause symmetry related structures to not be recognized by the program. Equivalent structures with different atom indexing (for instance due to rotations of *t*-butyl or phenyl groups) can also lead to ineffective filtering, unless graph isomorphisms are considered.^62^

“Interconversion error”, the error associated with not distinguishing and properly treating interconvertible and noninterconvertible structures, is subtler and trickier to process. On the potential energy surface, interconvertibility between TSs (first order saddle points) is governed by temperature-dependent barrier heights (second order saddle points), which are hard to characterize^63^ but have been shown to affect reaction dynamics.^64−67^ Clearly, two TSs differing only by, for instance, a small rotation of a C–Ph single bond (i.e., rotamers) are connected by a negligibly small energetic barrier, making these species easily interconvertible as they readily adopt the most energetically favorable structure to bypass the TS. On the other hand, conformationally locked structures, such as *C*_2_-symmetric biaryl moieties (Figure 1), cannot intercovert due to a high barrier associated with significant steric repulsion, and ergodicity is broken. Conformer generation tools are not necessarily bound by realistic kinetics, which results in the presence of different TS conformers within an ensemble that may not be accessible to one another. In principle, noninterconvertible TSs should be treated as separate reaction pathways, with the rate constants associated with each pathway leading to the same product being summed. On the other hand, interconvertible TSs should be treated as a single pathway. Improper treatment resulting in “double counting” in this setting would lead to an artificial decrease in the effective activation energy. Note that differentiating between conformational isomers and rotamers is a recurrent challenge in automated reaction network exploration.^11,12,68−71^

To illustrate how these error sources quantitatively impact selectivity predictions, we examine the *N*-methylation of a tropane (**1**) with isotopically labeled ^14^CH_3_I (Figure 2).^72−75^ Two conformations of the system (**1a** and **1b**) exist in equilibrium through a pyramidal inversion of the bridging nitrogen (**TSi**). An *S*_*N*_2 reaction with ^14^CH_3_I leads to two methylated isotopomers (**2a** and **2b**) formed through **TSa** and **TSb**, respectively. As the activation energies associated with the *S*_*N*_2 reaction are significantly larger (>12 kcal/mol) than that of the pyramidal N-inversion (<5 kcal/mol through **TSi**), the system operates under Curtin-Hammett conditions and the product distribution exclusively depends on the free energy barriers of **TSa** and **TSb**, independently of the energy of **1a** and **1b**.^76^ Using this system as an illustrative model, we calculate selectivity (expressed as an isotopomer ratio), employing different strategies to account for repeated and (non)interconvertible conformer issues.

**Figure 2:**
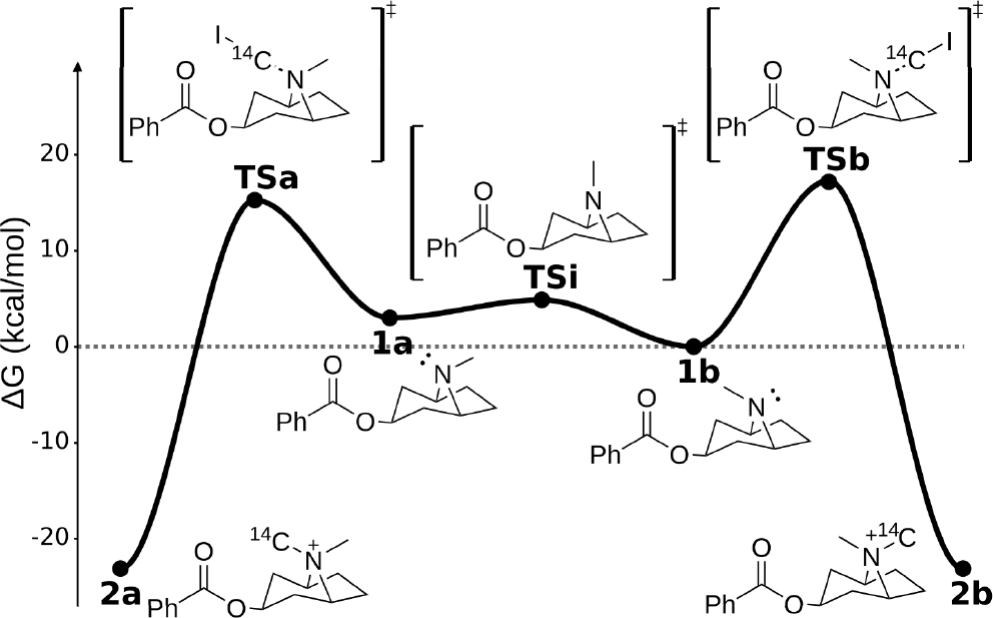
Free energy profile of the N-methylation reaction of 3-(benzoyloxy)-8-methyl-8-azabicyclo[3.2.1]octane with isotopically labeled ^14^CH_3_I via *S*_*N*_2 transition states **TSa** and **TSb**.

*Selectivity Prediction without Conformational Sampling*. As a first approximation, we identified structures for **TSa** and **TSb** which were optimized at the ωB97XD/def2-TZVP//ωB97XD/def2-SVP level (see Computational Details for additional information). These computations showed **TSa** to be 2.21 kcal/mol lower in energy than **TSb** (Figure 2). Taking
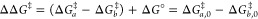
1where the 0 subscript indicates that both activation energies are taken with respect to the lowest energy intermediate (i.e., ΔΔ*G*^‡^ is expressed as the difference between the absolute free energies of **TSa** and **TSb**). In this case, ΔΔ*G*^‡^ = −2.21 kcal/mol and the major product at 298 K is predicted to be **2a** following
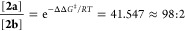
2as seen in Figure 3a.

**Figure 3:**
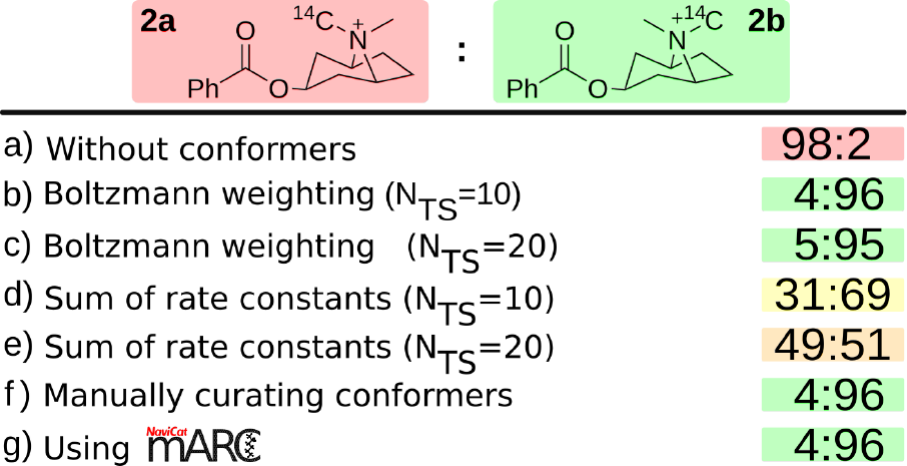
Isotopomer ratios were calculated with different strategies. (a) Without any conformational sampling. (b) Through Boltzmann weighting of the 10 lowest TS conformers per pathway. (c) As before, but considering the 20 lowest conformers per pathway. (d) Through addition of the rate constants from TS conformers, considering the 10 lowest TS conformers per pathway. (e) As before, but considering the 20 lowest conformers per pathway. (f) Calculated by manually examining and filtering the relevant TS conformers. (g) Calculated considering structures selected by *marc*.

*Selectivity Prediction with Conformational Sampling*. Figure 2 shows that the energies of **TSa** and **TSb** lie close to one another. Structurally, however, both the freely rotating benzoyloxy group and the 8-membered bicycle can adopt a multitude of orientations in the TS. To refine the prediction of selectivity, we performed a constrained conformational search of both **TSa** and **TSb** using CREST version 2.11,^50^ (note that we use CREST, as opposed to other programs,^77^ simply based on its popularity) keeping the relevant I–CH_3_–N distances fixed to ensure facile geometric convergence during subsequent ωB97XD reoptimizations. The resulting TS ensembles contained 86 (**TSa**) and 146 (**TSb**) structures (see Figure 4a/b for the superimposed structures). We now examine selectivity determined using Boltzmann weighting and summed rate constant approaches.

**Figure 4:**
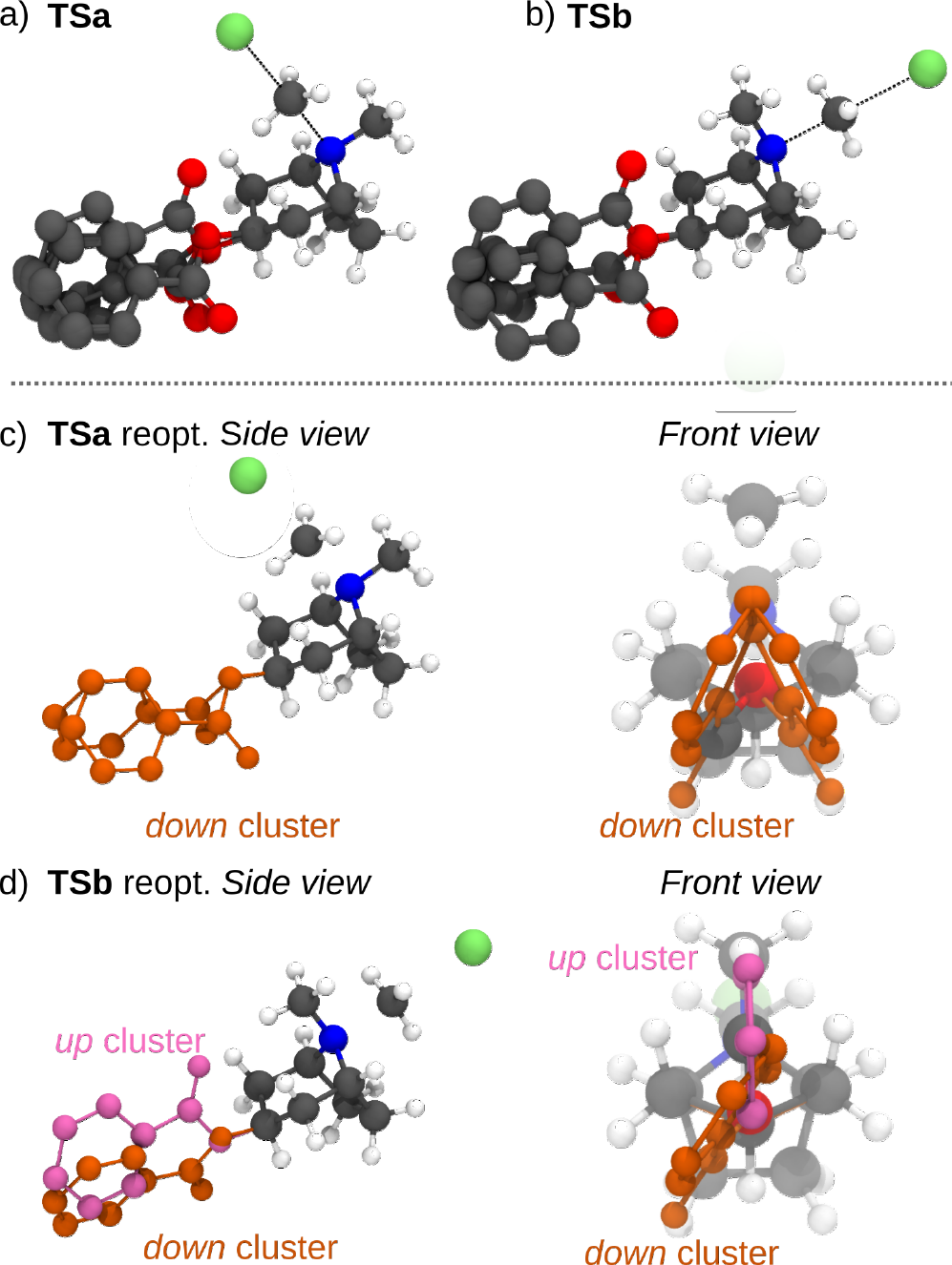
Superimposition of transition state conformers based on the RMSD of the tropane moiety. Full conformational ensemble of (a) **TSa** (86 structures) and (b) **TSb** (146 structures). (c) Twenty lowest energy conformers of **TSa** after DFT reoptimization, where all conformers belong to the same (orange) cluster. (d) Twenty lowest energy conformers of **TSb** after DFT reoptimization, where conformers belong to two clusters (downward pointing C=O, orange, or upward pointing C=O (pink).

*Boltzmann Weighting of Transition State Conformers*. Boltzmann weighting treats TSs as ensembles in which all conformers leading to a specific product are assumed to be freely interconvertible. As a first assumption, we took the *N*_*TS*_ = 10 lowest energy TS structures leading to each product (as predicted by their GFN2-xTB^78^ energies). The TS geometries were reoptimized at the ωB97XD/def2-TZVP//ωB97XD/def2-SVP level (see Computational Details for more information), during which some GFN2-xTB conformers converged to identical TSs (and some similar conformers diverged to different TSs, *vide infra*). We use Δ*G*_*j*,0_^‡^ to refer to the individual TS energies (relative to **1b**) and add a second subscript to differentiate **a** from **b** when necessary. Boltzmann weighting^79^ the ωB97XD TS energies of the 10 lowest energy **TSa** and **TSb** conformers gives the ensemble energies (indicated by the *ens*. superscript which is followed by the number of transition states included, *N*_*TS*_) as
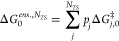
3where
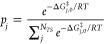
4are the Boltzmann weights. As per eq 3, Δ*G*_0_^*ens*^ is always higher than the lowest Δ*G*_*j*,0_. The weighting process is conducted separately for all **TSa** and **TSb** conformers.^80^ Substituting these values back into eq 2 yields
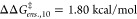
5and
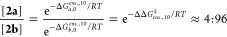
6as seen in Figure 3b. Thus, Boltzmann weighting reverses selectivity from the single conformer result (98:2, Figure 3). Of course, using the lowest 10 energy TS conformers leading to each product is arbitrary as we do not know the “true” number of unique pathways prior to conformer generation. If we assume 20 conformers to be a better choice and repeat the same process by reoptimizing the *N*_*TS*_ = 20 lowest energy TS structures, we obtain ΔΔ*G*_*ens*,20_^‡^ = 1.75 kcal/mol, giving a 5:95 product ratio (Figure 3d).

Recall that once the lowest energy TS is found, each additional TS conformer identified increases selectivity toward the *opposite* product. As an example, the highest energy **TSb** conformer in our ensemble lies 17.5 kcal/mol above **1b**, which is higher than all **TSa** conformers. If duplicates of this high energy conformer are mistakenly added to the ensemble, Δ*G*_*b*,0_^*ens*^ (which is normally lower than Δ*G*_*a*,0_^*ens*^) would slowly tend toward 17.5 kcal/mol, eventually reversing the predicted preferred product. For this reason, duplicate TSs can be problematic, leading to inaccurate selectivity predictions.

*Summing Rate Constants of Transition State Conformers*. Boltzmann weighting assumes free interconvertability of all TSs leading to a specific product. However, if this interconversion is precluded by a high energetic barrier, then these TSs are best characterized as belonging to different reaction valleys that proceed *in parallel* toward their products. Assuming this is the case for all TS conformers, the effective rate constant *k*_*eff*._ is given as
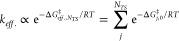
7where the mole fraction of the reference state (**1b**) is neglected for simplicity^39^ and −Δ*G*_*eff*_ is the effective activation energy toward the product. In other words, the effective kinetic rate constant is now the sum of all individual rate constants for all pathways leading to a specific product. In this case, the product distribution is the ratio of the total rate constants in each direction, given by
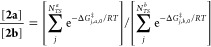
8which is analogous to eqs 1 and 2 with effective activation energies. Taking *N*_*TS*_^*a*^ = *N*_*TS*_^*b*^ = 10 and substituting in eq 8, we obtain ΔΔ*G*_*eff*,10_^‡^ = 0.48 kcal/mol and a product ratio [**2a**]/[**2b**] = 0.44, slightly selective toward **2b** (31:69, Figure 3d). Using *N*_*TS*_^*a*^ = *N*_*TS*_^*b*^ = 20 gives ΔΔ*G*_*eff*,20_^‡^ = 0.07 kcal/mol, corresponding to no selectivity (49:51, Figure 3e).

Here, additional low energy conformers significantly accelerate the reaction rate by providing multiple parallel pathways toward the product, while higher energy conformers do not influence the rate. Thus, “double counting” of low energy conformers in this setting lowers the effective activation energy in eq 7 by adding extra terms. In an extreme case, double counting of the lowest energy TS will give a *RTln*(2) lower barrier. Importantly, this treatment fundamentally differs from Boltzmann weighting, where high energy conformers would decelerate the reaction.

From the same sets of TS ensembles, we have obtained selectivity predictions ranging from highly selective for **2a** to highly selective for **2b**, solely by postprocessing the same results in different ways. To obtain unbiased selectivity predictions, conformer ensemble sorting and selection are required to differentiate interconvertible and parallel TS structures, which obtain accurate results.

*The Right Answer for the Right Reasons*. As highlighted above, we lack information about the ability of the different TS conformers in our ensemble to freely interconvert, because they are generated without relying on any energetic criterion. For the exemplary case studied here, however, it is possible to manually sort the conformers to arrive at the correct selectivity. Inspecting the DFT-reoptimized **TSa** ensemble reveals only a single conformer family in which all structures are freely interconvertible. This family has a Δ*G*_*a*,0,*down*_^‡^ ≈ 15.3 kcal/mol above **1b** and is characterized most notably by a downward pointing C=O bond (Figure 4c). In contrast, the **TSb** ensemble consists of two distinct conformer families characterized by either upward or downward pointing C=O bonds (Figure 4d), having values of Δ*G*_*b*,0,*up*_^‡^ ≈ 17.5 kcal/mol and Δ*G*_*b*,0,*down*_^‡^ = 13.4 kcal/mol, respectively.^81^ Moving between these two clusters requires overcoming second order saddle points with non-negligible barriers of over 8 kcal/mol above the TSs. As a result, all TSs can be grouped into one of three clusters: either **TSa** with a downward pointing C=O bond, **TSb** with a downward pointing C=O bond, or **TSb** with an upward pointing C=O bond. Using eq 3 we obtain the ensemble energies for each cluster, which can then be separately added, as in eq 7, to calculate the final selectivity using eq 8. Doing so gives [**2a**]/[**2b**] ≈ 4:96 (Figure 3f). In the end, only three (of the initial 212) TS conformers actually dictate the selectivity of the reaction. Note that this result is in agreement with the expected reactivity of tropanes, which typically privilege attacks from the less encumbered *Re*-side.^82,83^

*marc: An Automated Clustering Tool to Avoid Errors*. Manually curating structures is time-consuming and unsuitable for all but the simplest systems. To automate this process, we developed *marc* (***m***odular ***a***nalysis of ***r***epresentative ***c***onformers) as a simple command line tool to process conformational ensembles. For a given ensemble, independent of its origin, *marc* uses a mix of geometric (both symmetry-informed heavy-atom RMDSs and dihedral angles) and energetic (if available) information to perform clustering designed to obtain an optimal number of structures needed to completely cover the conformational space. The general workflow of *marc* is shown in Figure 5.

**Figure 5:**
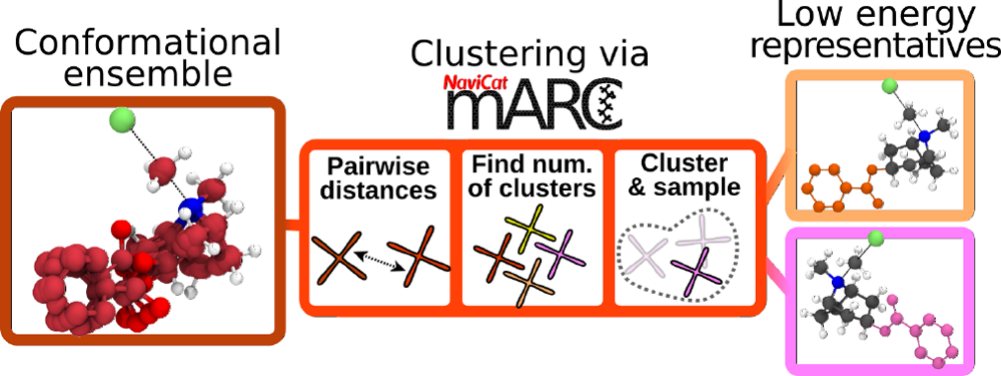
Workflow of *marc*. From a conformer ensemble, *marc* computes pairwise distances using different metrics (heavy atom RMSDs, relative energies, and dihedral angles) to construct a compound distance matrix, finds the optimal number of clusters using the silhouette method and k-means clustering, and samples the lowest energy structures belonging to each cluster.

Applying *marc* (using the default settings) to the (*N*_*TS*_^*a*^ = 20) DFT-refined ensembles gives a single cluster containing all conformers. The largest heavy-atom RMSD between two structures is a non-negligible 0.87 Å, which is sufficiently large to be considered as unique structures based on simple RMSD filtering using predefined thresholds. On the other hand, the maximum energy difference is only 0.01 kcal/mol. By combining energetic and structural criteria, *marc* successfully identifies all **TSa** conformers as being interconvertible. In contrast, the (*N*_*TS*_^*b*^ = 20) ensemble is found to have two clusters. The first contains only a single structure with a downward pointing C=O bond while the second contains the 19 other structures having upward pointing C=O bonds. Here, the maximum heavy-atom RMSD among the conformers is slightly larger (1.32 Å vs 0.87 Å) while the maximum energy difference is 3.2 kcal/mol with the lowest energy conformer belonging to the first cluster and 19 higher energy conformers belonging to the second. As *marc* selects the same conformers found using manual inspection in the previous section, we once again obtain an isotopomer ratio of 4:96 (Figure 3g).

The above results were obtained by processing the 20 lowest energy conformers for both **TSa** and **TSb** optimized at the DFT level. This raises an important question: could the 40 DFT reoptimizations have been completely avoided and the same results obtained? Running *marc* directly on the full CREST-generated ensembles dramatically reduces the number of computations needed. For the original 86 structure **TSa** ensemble, three clusters populated by 1, 1, and 84 structures are identified. The largest corresponds to the downward pointing C=O bond species discussed earlier, while two others contain species with upward pointing C=O bonds that are significantly higher in energy (such that they were not included in the 20 lowest energy structures selected for DFT refinement).^84^ For the 146-structure **TSb** ensemble, two clusters characterized by downward (101 structures) and upward pointing C=O bonds (45 structures) are found. If just five total TS structures (one structure from each of the three **TSa** and two **TSb** clusters) are reoptimized by using DFT, a 6:94 product ratio is found. This closely matches the 4:96 product ratio obtained by processing the 20 lowest energy DFT reoptimized conformers from **TSa**/**TSb**, at ∼1/8 of the computational cost.^85^ In short, *marc* also simplifies the postprocessing of conformational ensembles that have not yet been refined with DFT, reducing large ensembles to a handful of representative structures thereby, reducing computational expense. Such savings become increasingly important when the CHCS protocol is applied to large species and/or in high-throughput settings.

In conclusion, using the N-methylation of tropane with isotopically labeled ^14^CH_3_I leading to two isotopomers as a model reaction, we have shown how an ensemble of the same TS conformers can be processed in different ways to obtain any possible selectivity prediction under Curtin-Hammett conditions. These different selectivity predictions arise from errors associated with the presence of “repeated conformer” and “interconversion” errors associated with distinguishing when various TSs are freely able to interconvert among themselves. We then introduced *marc*, a simple command line tool designed to analyze conformational ensembles and select the most representative structures. Using *marc*, accurate predictions of selectivity can be obtained with a significantly reduced computational cost. Having identified the problem, as well as a path toward a solution, a more thorough benchmark of clustering and conformational ensemble processing methodologies across different systems will be the subject of future work.

## Computational Details

Conformer ensemble generation was performed with CREST^49,50,78^ version 2.11 using the default settings except doubling the default metadynamics runtime and setting a 0.5 au harmonic constraint placed on the I–CH_3_–N atoms involved in the *S*_*N*_2 transition state. Selected geometries were optimized at the ωB97XD^86^/def2-SVP^87^ level of theory as implemented in Gaussian 16.^88^ Vibrational frequency analysis was used to confirm that stationary points were either minima (no imaginary frequencies), transition states (one imaginary frequency), or second order saddle points (two imaginary frequencies) on the potential energy surface. Refined energy estimates were obtained by single point computations at the ωB97XD/def2-TZVP level on ωB97XD/def2-SVP geometries. Free energy corrections were taken from the ωB97XD/def2-SVP computations using the GoodVibes program.^38^ Solvent effects were included in the single point computations using the SMD^89^ implicit solvation model (for acetonitrile). Reported free energies include the solvation-corrected ωB97XD/def2-TZVP electronic energies, and the ωB97XD/def2-SVP free energy corrections. All structures and computed energies are available in the “examples” directory of https://github.com/lcmd-epfl/marc.

*marc* and user instructions can be found at https://github.com/lcmd-epfl/marc with DOI 10.5281/zenodo.12569985. Clustering is performed using the k-means algorithm, with a multidimensional scaling of the averaged dissimilarity matrices as input, as implemented in the scikit-learn python library.^90^ The silhouette score method is used to assess the number of clusters. RMSDs are computed considering all isomorphisms between the molecular graphs, which accounts for molecular symmetry.^62^*marc* also allows users to use other clustering algorithms and dissimilarity matrix types, including agglomerative methods and dihedral angles as pioneered by the CENSO program.^79^

## Supporting Information Available

Transparent Peer Review report available (PDF)
