# Supplementary material for: Overcoming the Pitfalls of Computing Reaction Selectivity from Ensembles of Transition States
Source: J Phys Chem Lett. 2024 Jul 11;15(29):7363–70. doi: 10.1021/acs.jpclett.4c01657 (PMC11284845; doi:10.1021/acs.jpclett.4c01657)
Supplement: Supplementary file 1 — jz4c01657_si_001.pdf [file jz4c01657_si_001.pdf]

jz-2024-01657g.R1

Name: Peer Review Information for "Overcoming the Pitfalls of Computing Reaction Selectivity from Ensembles of Transition States"

First Round of Reviewer Comments

Reviewer: 1

Comments to the Author

In the letter 'Overcoming the Pitfalls of Computing Reaction Selectivity from Ensembles of Transition States' jz-2024-01657g the authors concisely discuss implications of conformational sampling on the calculation of reaction kinetics via transition state theory.

Overall the authors do an excellent job in this. All calculations follow best computational practices, the examples shown are meaningful and relevant. The letter provides a nice overview over problems faced in computing reaction pathways for flexible systems and presents a straight-forward strategy to address these issues.

I think the manuscript can be accepted without (much) changes.

Some comments that the authors may want to consider in a fully optional revision:

1. I believe what makes marc quite powerful is the consideration of molecular graph isomorphism in the RMSD computation. This likely is one advancement the author's program has over, for example, the conformational sorting algorithm in crest. I think they should mention this more prominently, rather than just at the very end of the manuscript.

2. On page 2/8, l.36, the authors are likely referring to 'broken ergodicity' and overcoming it by conformational sampling/global optimization. It would be good to name this properly.

3. What the authors refer to as 'repeated conformer' would probably also fall under the plain definition of a rotamer, i.e., degenerate conformational minima on the PES that can be created by permutation of equivalent nuclei and/or local symmetry. I think the distinction with 'interconvertible structures' is important here, but given a sufficiently long pathway all of these structures are surely interconvertible? Overall 'permutational isomers' may be a more general nomenclature.

4. There is a whole body of work deidcated to similar problems of identifying equivalent isomers and their impact on reaction pathways that the authors are not citing, mainly in context of the rare events community and setting up kinetic transition/reaction networks. The key difference seems to be that the author's approach focuses on post-processing of conformational ensembles, while in

the kinetic transition network creation one attempts to address this during the growing the network. This might be worth looking into for future work.

Reviewer: 2

Comments to the Author

This is a DFT study of a quaternization of an N-alkaloid, calling special attention to the prediction of stereoselectivity in the resulting products. This example is used to illustrate some fundamental problems in the computational modelling of such selectivities. To have the underlying conformational sampling problem explained so clearly, and a potential automated tool presented to address it, is, in my opinion, a valuable contribution, and I commend the authors for the excellent scholarly presentation of the problem. I am inclined to recommend acceptance of the paper, but have a few reservations and suggestions that should be addressed in a revised version.

The authors picked a target reaction, methylation of an N-methyl compound, that is only stereoselective with isotope labelling. If I see correctly, such labelling experiments have not yet performed (at least I could find no mention in ref. [65]). I appreciate that this is not a key point for the present study (and that, for once, true predictions are reported), but it is a bit unfortunate that the results cannot be directly compared to experiment (the ultimate test). This should be pointed out clearly and at least some effort be made to relate the findings to what is known qualitatively for related systems (e.g. for methyl/benzyl derivatives, with the caveat of course that steric effects may be important if not overriding here).

I see that interconversion between TSs is a valid assumption within TST (if equilibria between minima and TSs are assumed, why not those between TSs and 2nd order saddle points), but chemically I need to be convinced that such higher order saddle points are really involved in chemical transformations. I would welcome some more discussion of this aspect with reference, e.g. to MD simulations (i.e. if such areas on the PES are really visited at all – not for the target reaction, but in general).

Seeing that a key part of the paper is the newly developed tool, marc, its working remains a bit nebulous. I would welcome some more details on the steps sketched in Fig. 5, e.g. how are the distance pairs, thresholds for the RMSDs etc. chosen? I appreciate much of this will be covered (one might also say, hidden) on the website provided, but that brings me to another point, namely long-time availability of published material. To have that in the hands of a private company, GitHub, may be common practice, but I'd be more comfortable if that could be accessed independently of that as well. Is there a possibility to deposit key material (code, example inputs and outputs) on a University repository, or submit as part of electronic SI on the publisher's website? At the very least,

a description of the program should be provided in sufficient detail that others could rewrite or implement it elsewhere.

And a methodological quibble: TSa and TSb involve charge separation, leading to ion pairs. The optimizations in the gas phase could lead to artifacts if the charge separation would be so far advanced that the structures would become very sensitive to a polar environment (in the worst of cases, this could prevent location of a TS altogether – apparently not the case here). Again, a comment on this issue would be in order (and perhaps a few exploratory calculations involving optimizations in a continuum could be shown in an ESI).

With some additional discussion and documentation along these lines, I am sure the paper will improve further and be entirely suitable for publication.

Reviewer: 3

Comments to the Author

In the manuscript "Overcoming the Pitfalls of Computing Reaction Selectivity from Ensembles of Transition States" by Laplaza, Wodrich, and Corminboeuf, the aspects of conformational sampling of transition state geometries are discussed. Furthermore, a tool is presented to automatically prune conformational ensembles. The paper is well-written, but in its current form not publishable for JPCL:

A. The authors highlight an important point of artificial double counting of conformers as a result from flawed conformer generation and pruning algorithms. This is a broader problem, which appears to be particularly severe for the calculation of reaction rates. What seems to be missing from the current discussion is the effect of the substrate (minimum) ensembles, i.e. 1a and 1b. It appears to me that this is particularly important and can further influence in a positive or in a bad way the computed rates. Particularly, for the additive reaction rate scheme discussed in the later part of the manuscript. In fact, the aspect of interconvertibility must be discussed in that context as well. One might think that every TS conformer has only one specific minimum conformer that can be obtained from the IRC. This discussion is still missing in the manuscript entirely.

B. The main issue is that the authors combine an interesting case study, nicely demonstrating the difficulty of using TS ensembles with a method paper on pruning conformer ensembles. On the one hand, the JPCL communication format is a bit short for method details, but I must say that the authors don't give any information on how the "marc" tool is working - this description must not be outsourced entirely to the description in the github repo. On the other hand, they are discussing a

single case here. It may be an emblematic one, but that is certainly not enough for presenting a method/tool that supposingly can be used for conformer ensembles of a broad range of molecules. Hence, it seems that marc should be published elsewhere with enough methodological details and benchmark results. The case study to highlight the difficulty of TS conformer sampling can then be published as communication as here in JPCL. If the authors manage to fit in all the methodological description + benchmarks for marc in here somehow, that might work as well. But frankly, this seems like two separate projects and papers to me, since marc does not appear to be limited to TS geometries. So a large benchmark study is necessary and, scince the authors used CREST, how does it compare to its built-in "-cregen" and "-cluster" functions to filter conformer ensembles?

C. As of now, the SI is missing. So structures are missing & the hand-selection of conformers is not easy to follow, how these were selected. I furthermore have the following specific remarks in the manuscript:

a. The authors should comment on where the 10 and 20, resp., structures come from (e.g. in Fig. 3). How were they chosen. CREST was used for conformer generation but you can basically "tune" the relative rates much by selecting specific conformers.

b. P.3, l.7/8: I disagree with the statement that "the method [...] used for conformer generation is arbirtrary" w.r.t. the specific problem. CREST, working in 3D Cartesian space and being based on dynamics is clearly more prone to generating identical structures with redundant information than, let's say, a rule-based conformer search algorithm that searches for the lowest conformer on a grid of torsion angles. In the latter case, filtering should be required to a much lower degree.

c. P.3 Fig. 4a/b: this does not look like an overlay of 86 and 146, resp., structures at all. Maybe the authors can comment on what is plotted here exactly or are the structures that similar? If so, I am surprised that the "-cregen" function in CREST does not do a better job in filtering.

Author's Response to Peer Review Comments:

Manuscript ID: jz-2024-01657g

Title: "Overcoming the Pitfalls of Computing Reaction Selectivity from Ensembles of Transition States"

Author(s): Laplaza, Ruben ; Wodrich, Matthew; Corminboeuf, Clémence

---

Dear Editor,

Please find attached the revised version of our manuscript. We are pleased the reviewers thought that the topic of the manuscript was interesting and have suggested revision that will improve generally readability and understanding. Below, you will find our responses to the

points raised by the reviewers. Reviewer comments are shown in **black**, our responses in **blue**, and changes to the manuscript in **red**.

We hope that these corrections meet the expectations of the reviewers and we thank you for considering our revised version for publication.

With best wishes,

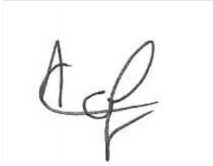

Clémence Corminboeuf

---

*Comments are in **black**, responses are in **blue**, and changes to the manuscript are in **red**.*

Reviewer 1:

Comment #1: I believe what makes marc quite powerful is the consideration of molecular graph isomorphism in the RMSD computation. This likely is one advancement the author's program has over, for example, the conformational sorting algorithm in crest. I think they should mention this more prominently, rather than just at the very end of the manuscript.

The use of molecular graph isomorphism, as well as its practical implementation for RMSD, were pioneered by Meli and Biggin [*J. Cheminform.* **2020**, *12*, 49]. While we do not want to overshadow their contribution, we have now raised the point earlier in the manuscript.

Equivalent structures with different atom indexing (for instance due to rotations of *t*-butyl or phenyl groups) can also lead to ineffective filtering, unless graph isomorphisms are considered.[62]

Comment #2: On page 2/8, l.36, the authors are likely referring to 'broken ergodicity' and overcoming it by conformational sampling/global optimization. It would be good to name this properly.

We have adjusted the text to explicitly mention “broken ergodicity.”

[...] cannot intercovert due to a high barrier associated with significant steric repulsion, and ergodicity is broken.

Comment #3: What the authors refer to as 'repeated conformer' would probably also fall under the plain definition of a rotamer, i.e., degenerate conformational minima on the PES that can be created by permutation of equivalent nuclei and/or local symmetry. I think the distinction with 'interconvertible structures' is important here, but given a sufficiently long pathway all of these structures are surely interconvertible? Overall 'permutational isomers' may be a more general nomenclature.

Since the definition of a “repeated conformer” is context dependent (primarily through time and temperature), respectively, we believe that our general terminology is better suited.

Comment #4: There is a whole body of work deidcated to similar problems of identifying equivalent isomers and their impact on reaction pathways that the authors are not citing, mainly in context of the rare events community and setting up kinetic transition/reaction networks. The key difference seems to be that the author's approach focuses on post-processing of conformational ensembles, while in the kinetic transition network creation one attempts to address this during the growing the network. This might be worth looking into for future work.

We thank the reviewer for pointing this out. We added a statement and the relevant citations related to work addressing these points in the field of reaction network exploration.

Note that differentiating between conformational isomers and rotamers is a recurring challenge in automated reaction network exploration.[11,12,68-71]

Reviewer 2:

Comments:

Comment #1: The authors picked a target reaction, methylation of an N-methyl compound, that is only stereoselective with isotope labelling. If I see correctly, such labelling experiments have not yet performed (at least I could find no mention in ref. [65]). I appreciate that this is not a key point for the present study (and that, for once, true predictions are reported), but it is a bit unfortunate that the results cannot be directly compared to experiment (the ultimate test). This should be pointed out clearly and at least some effort be made to relate the findings to what is known qualitatively for related systems (e.g. for methyl/benzyl derivatives, with the caveat of course that steric effects may be important if not overriding here).

We have better contextualized the results, which are in agreement with chemical intuition and previous experimental findings on similar systems.

Note that this result is in agreement with the expected reactivity of tropanes, which typically privilege attacks from the less encumbered *Re*-side.[82,83]

Comment #2: I see that interconversion between TSs is a valid assumption within TST (if equilibria between minima and TSs are assumed, why not those between TSs and 2nd order saddle points), but chemically I need to be convinced that such higher order saddle points are really involved in chemical transformations. I would welcome some more discussion of this aspect with reference, e.g. to MD simulations (i.e. if such areas on the PES are really visited at all – not for the target reaction, but in general).

We have added several references discussing the effect of higher order saddle points in reaction dynamics.

On the potential energy surface, interconvertibility between TSs (1st order saddle points) is governed by temperature-dependent barrier heights (2nd order saddle points), which are hard to characterize[63] but have been shown to affect reaction dynamics.[64-67]

Comment #3: Seeing that a key part of the paper is the newly developed tool, marc, its working remains a bit nebulous. I would welcome some more details on the steps sketched in Fig. 5, e.g. how are the distance pairs, thresholds for the RMSDs etc. chosen? I appreciate much of this will be covered (one might also say, hidden) on the website provided, but that brings me to another point, namely long-time availability of published material. To have that in the hands of a private company, GitHub, may be common practice, but I'd be more comfortable if that could be accessed independently of that as well. Is there a possibility to deposit key material (code, example inputs and outputs) on a University repository, or submit as part of electronic SI on the publisher's website? At the very least, a description of the program should be provided in sufficient detail that others could rewrite or implement it elsewhere.

We have deposited the version of *marc* used in the paper in a Zenodo entry, which ensures long term preservation and provides a unique DOI (10.5281/zenodo.12569985). This includes the *marc* tutorials and instructions that are included with the package.

In addition, we have added more details into the Figure 5 caption.

Workflow of *marc*. From a conformer ensemble, *marc* computes pairwise distances using different metrics (heavy atom RMSDs, relative energies, and dihedral angles) to construct a compound distance matrix, finds the optimal number of clusters using the silhouette method and k-means clustering, and samples the lowest energy structures belonging to each cluster.

Comment #4: And a methodological quibble: TSa and TSb involve charge separation, leading to ion pairs. The optimizations in the gas phase could lead to artifacts if the charge separation would be so far advanced that the structures would become very sensitive to a polar environment (in the worst of cases, this could prevent location of a TS altogether – apparently not the case here). Again,

a comment on this issue would be in order (and perhaps a few exploratory calculations involving optimizations in a continuum could be shown in an ESI).

We agree that solvation could play a role in this reaction, although reactions of this type are often conducted in non-polar solvents such as toluene (which has been better clarified in the text). Moreover, the goal of this work is less focused on the accurate modeling of this specific reaction and more on the general pitfalls of computing selectivity, regardless of the specific reaction.

We have added this precision to the manuscript:

Due to their ion-pair character, the energies of **TSa** and **TSb** may be dependent on the solvent employed. Here, we have carried out our computations in the gas phase for demonstrative purposes.

Reviewer 3:

Comments:

Comment #1: The authors highlight an important point of artificial double counting of conformers as a result from flawed conformer generation and pruning algorithms. This is a broader problem, which appears to be particularly severe for the calculation of reaction rates. What seems to be missing from the current discussion is the effect of the substrate (minimum) ensembles, i.e. 1a and 1b. It appears to me that this is particularly important and can further influence in a positive or in a bad way the computed rates. Particularly, for the additive reaction rate scheme discussed in the later part of the manuscript. In fact, the aspect of interconvertibility must be discussed in that context as well. One might think that every TS conformer has only one specific minimum conformer that can be obtained from the IRC. This discussion is still missing in the manuscript entirely.

Following the assumption that the system operates under Curtin-Hammett conditions (as stated in the manuscript), any conformational changes of the intermediates preceding the TS have negligible barriers compared to the reaction of interest. We have further clarified this point in the manuscript.

As the activation energies associated with the S_N2 reaction are significantly larger (>12 kcal/mol) than that of the pyramidal N-inversion (<5 kcal/mol through **TSi**), the system operates under Curtin-Hammett conditions and the product distribution exclusively depends on the free energy barriers of **TSa** and **TSb**, independently of the energy of **1a** and **1b**.

Comment #2: The main issue is that the authors combine an interesting case study, nicely demonstrating the difficulty of using TS ensembles with a method paper on pruning conformer ensembles. On the one hand, the JPCL communication format is a bit short for method details, but I must say that the authors don't give any information on how the "marc" tool is working - this description must not be outsourced entirely to the description in the github repo. On the other hand, they are discussing a single case here. It may be an emblematic one, but that is certainly not enough for presenting a method/tool that supposingly can be used for conformer ensembles of a broad range of molecules. Hence, it seems that marc should be published elsewhere with enough methodological details and benchmark results. The case study to highlight the difficulty of TS conformer sampling can then be published as communication as here in JPCL. If the authors manage to fit in all the methodological description + benchmarks for marc in here somehow, that might work as well. But frankly, this seems like two separate projects and papers to me, since marc does not appear to be limited to TS geometries. So a large benchmark study is necessary and, scince the authors used CREST, how does it compare to its built-in "-cregen" and "-cluster" functions to filter conformer ensembles?

The goal of this work is focused on bringing attention to the pitfalls of transition state conformer sampling and highlighting how filtering and pruning tools can be used to avoid them. To this end, we aimed to make the manuscript succinct and easy to read by providing a simple, yet informative, sample problem as well as a brief demonstration of how marc aids in solving this problem.

Having identified the problem as well as a path towards a solution, we fully agree that a more thorough benchmark of clustering and conformational ensemble processing methodologies across

different systems should be undertaken. Indeed, we plan to make this the subject of future work (which is now indicated in the main text).

Having identified the problem, as well as a path towards a solution, a more thorough benchmark of clustering and conformational ensemble processing methodologies across different systems will be the subject of future work.

Comment #3: As of now, the SI is missing. So structures are missing & the hand-selection of conformers is not easy to follow, how these were selected.

All the structures are provided as xyz files in the github page of marc (https://github.com/lcmd-epfl/marc/tree/main/examples/tropanes_example) as well as in the corresponding Zenodo record (10.5281/zenodo.12569985). This is stated in the computational details section:

All structures and computed energies are available in the “examples” directory of https://github.com/lcmd-epfl/marc. [...] marc and user instructions can be found at https://github.com/lcmd-epfl/marc with DOI 10.5281/zenodo.12569985.

Comment #4: I furthermore have the following specific remarks in the manuscript: The authors should comment on where the 10 and 20, resp., structures come from (e.g. in Fig. 3). How were they chosen. CREST was used for conformer generation but you can basically “tune” the relative rates much by selecting specific conformers.

For the 10 conformer example, this is specified in the main text:

As a first assumption, we took the N_{TS} = 10 lowest energy TS structures leading to each product (as predicted by their GFN2-xTB[78] energies). The TS geometries were reoptimized at the ωB97XD/def2-TZVP//ωB97XD/def2-SVP level (see Computational Details for more information), during which some GFN2-xTB conformers converged to identical TSs (and some similar conformers diverged to different TSs, vide infra).

And has been further clarified when moving to 20 conformers per pathway:

[...] Of course, using the lowest 10 energy TS conformers leading to each product is arbitrary, as we do not know the “true” number of unique pathways prior to conformer generation. If we assume 20 conformers to be a better choice and repeat the same process by reoptimizing the N_{TS} = 20 lowest energy TS structures, we obtain [...]

Comment #5: P.3, l.7/8: I disagree with the statement that “the method [...] used for conformer generation is arbitrary” w.r.t. the specific problem. CREST, working in 3D Cartesian space and being based on dynamics is clearly more prone to generating identical structures with redundant information than, let’s say, a rule-based conformer search algorithm that searches for the lowest conformer on a grid of torsion angles. In the latter case, filtering should be required to a much lower degree.

We do not know whether CREST is more or less prone to generating identical conformers. CREST also includes several internal checks for duplicates, while rotamer libraries may generate duplicates due to symmetries/atom indexing, and may jump over unphysical barriers leading to interconversion errors. In any case, we have revised the main text to better convey our meaning.

(note that we use CREST, as opposed to other programs,[77] simply based on its popularity)

Comment #6: P.3 Fig. 4a/b: this does not look like an overlay of 86 and 146, resp., structures at all. Maybe the authors can comment on what is plotted here exactly or are the structures that similar? If so, I am surprised that the "-cregen" function in CREST does not do a better job in filtering.

The structures appear very similar when aligned by the 8-membered bicycle. Despite this, there is a non-negligble RMSD between the two structures (0.87 Å), which is noted in text (see below). Also, the cregen option of the recently released CREST 3.0 was not used - we only run default CREST 2.11 sampling.

The largest heavy-atom RMSD between two structures is a non-negligible 0.87 Å , which is sufficiently large to be considered as unique structures based on simple RMSD filtering using predefined thresholds. On the other hand, the maximum energy difference is only 0.01 kcal/mol.
